# *trans*-Di­aqua­bis­(pyridin-2-yl thio­phen-2-yl ketone-κ^2^*N*,*O*)nickel(II) bis­(tetra­fluorido­borate)

**DOI:** 10.1107/S2414314626002403

**Published:** 2026-03-11

**Authors:** Barry L. Westcott, Gary S. Nichol

**Affiliations:** a Central Connecticut State University, Department of Chemistry and Biochemistry 1615 Stanley St New Britain CT 06050 USA; b The University of Edinburgh School of Chemistry Joseph Black Building David Brewster Road Edinburgh EH9 3FJ United Kingdom; Vienna University of Technology, Austria

**Keywords:** crystal structure, nickel, pyridyl ketone, hydrogen bonding

## Abstract

The complex title salt, [Ni(C_10_H_7_NOS)_2_(H_2_O)_2_](BF_4_)_2_, has monoclinic symmetry (space group *P*2_1_/*c*) with the central Ni^II^ atom located at an inversion center. The coordination environment around the Ni^II^ atom is pseudo-octa­hedral with bonding to pyridyl N [2.0342 (6) Å] and carbonyl O [2.0402 (5) Å] atoms from two chelating 2-thienyl 2-pyridyl ketone ligands. The remaining bonds are to water mol­ecules [2.0989 (7) Å], which are hydrogen-bonded to BF_4_^−^ counter-ions. The crystal structure was refined using nonspherical scattering factors.

## Abstract 2

The Ni^II^ atom in the complex cation of the title salt is located on an inversion center and shows a pseudo-octa­hedral coordination with two ligands bound through the carbonyl oxygen and pyridyl nitro­gen atoms in the equatorial and two aqua ligands in the axial positions.

## Graphical abstract

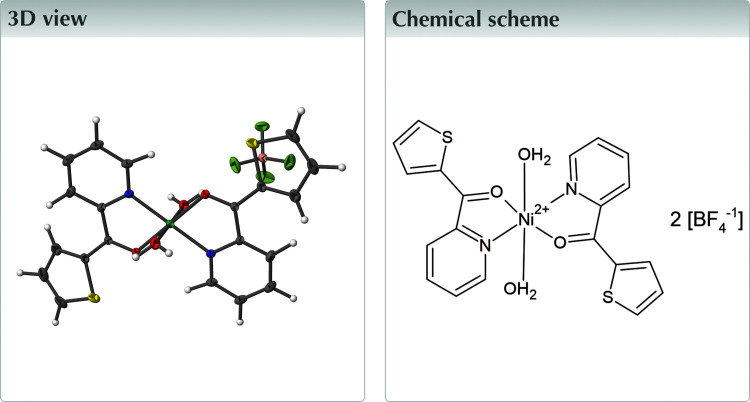

## Structure description

The ligand di-2-pyridyl ketone (dpk) displays an unusual hydration reaction in the presence of a metal ion allowing this ligand to have three distinct binding sites for metals – the two pyridyl nitro­gen atoms and the diol (Efthymiou *et al.*, 2010 ▸; Stamatatos & Christou, 2009 ▸). Derivatives of dpk, such as di-2-pyridyl ketone oxime (dpko), do not undergo this hydration reaction but can still form multiple binding sites with metals (Stoumpos *et al.*, 2010 ▸). The structurally related ligand 2-thienyl-2-pyridyl ketone (tpk) changes the donor of one ring from N to S, which affects the overall electronic and structural capabilities of the compound. To date, only one metal complex with the tpk ligand has been reported (Sommerer *et al.*, 1998 ▸). In this complex, only the pyridyl N – not the thienyl S – bonds with the metal ion, and the ketone remains unhydrated despite the presence of water.

**Figure 1:**
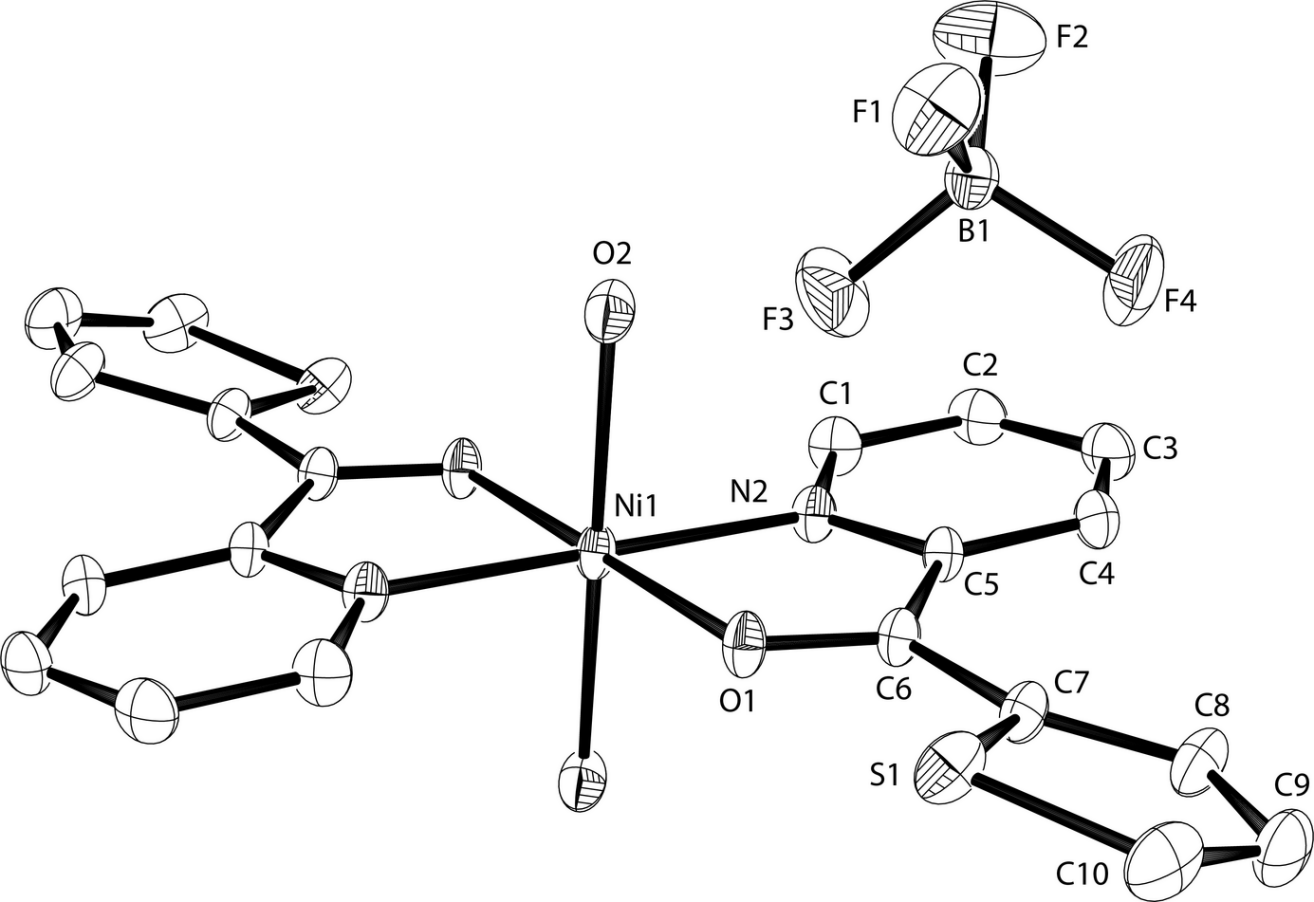
The mol­ecular structures of cation and anion in the complex title salt. Displacement ellipsoids are drawn at the 50% probability level; only atoms of the asymmetric unit are labeled and H atoms omitted for clarity.

The complex title salt (Fig. 1 ▸) is structurally and crystallographically similar to the previously reported Cu^II^ analog (Sommerer *et al.*, 1998 ▸). The Cu^II^ complex salt was refined in the alternate space group of *P*2_1_/*n* (*versus P*2_1_/*c*) and can be considered as isoconfigurational (Lima de Faria *et al.*, 1990 ▸). Both the Ni^II^ atom of the current structure and the Cu^II^ atom from the previous report sit on an inversion center with pseudo-octa­hedral coordination. The tpk ligands coordinate through pyridyl N [2.0342 (6) Å] and carbonyl O [2.0402 (5) Å] atoms in the equatorial plane and through oxygen atoms from water mol­ecules in the axial positions, leading to an [O_4_N_2_] coordination set. The Ni—O distances with the aqua ligands are significantly shorter than in the Cu^II^ complex [2.0989 (7) Å *versus*. 2.409 (3) Å], likely due to the Jahn–Teller distortion seen for octa­hedral Cu^II^ complexes (Procter *et al.*, 1968 ▸).

All other bond lengths and angles are consistent with the previously reported Cu^II^ complex (Sommerer *et al.*, 1998 ▸) and Ni^II^ complexes with similar ligands, such as di-2-pyridyl ketone (Sue-Lein *et al.*, 1986 ▸) or di-2-pyridyl ketone oxime (Stamou *et al.*, 2025 ▸).

**Figure 2:**
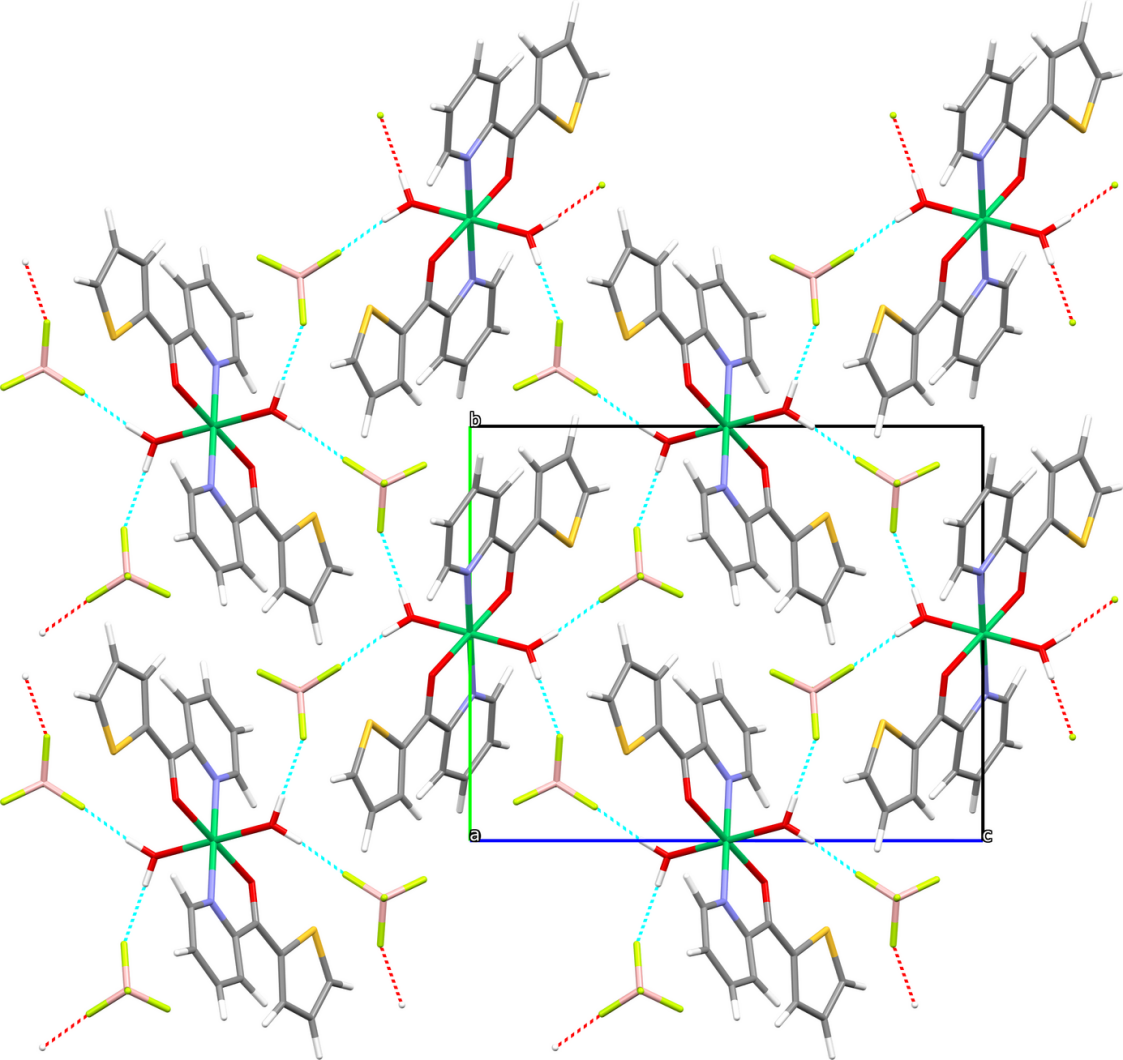
The crystal structure of the complex title salt in a view along the *a* axis. O—H⋯F hydrogen-bonding is shown by dashed lines.

The BF_4_^−^ anion acts as a hydrogen-bonding acceptor with the coordinating water mol­ecules as donors (Table 1 ▸, Fig. 2 ▸). These medium–strong O—H⋯F inter­actions link adjacent complexes and anions together to form a hydrogen-bonded sheet, which propagates in the *bc* plane.

## Synthesis and crystallization

Ni(BF_4_)_2_·6H_2_O and aceto­nitrile were used as received from Thermo-Fisher; 2-thienyl 2-pyridyl ketone was used as received from Rieke Metals. [Ni(C_10_H_7_NOS)_2_(H_2_O)_2_](BF_4_)_2_ was synthesized following a literature procedure (Sommerer *et al.*, 1998 ▸): excess Ni(BF_4_)_2_·6H_2_O (0.2294 g, 0.675 mmol) was combined with 2-thienyl 2-pyridyl ketone (0.1997 g, 1.10 mmol) in 35 ml of aceto­nitrile at room temperature affording a dark green solution, which was allowed to slowly evaporate until production and isolation of dark-green crystals suitable for X-ray diffraction (40 d).

## Refinement

Crystal data, data collection and structure refinement details are summarized in Table 2 ▸. The crystal used for data collection was twinned. Non-merohedral twinning was handled with *CrysAlis PRO* (relation between the twin domains by matrix [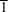 0 0 / 0 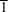 0 / 0.9467 0 1]) and refined to a twin scale factor of 0.4990 (6). The crystal structure was refined using nonspher­ical scattering factors, with all atoms refined anisotropically by using the ‘final’ default settings of *NoSpherA2* (Kleemiss *et al.*, 2021 ▸); *ORCA 5.0* (Neese, 2022 ▸) was used for quantum mechanical calculations. The latter programs are implemented in *OLEX2* (Dolomanov *et al.*, 2009 ▸). The _olex2_refinement_description section of the CIF gives further details.

## Supplementary Material

Crystal structure: contains datablock(s) I. DOI: 10.1107/S2414314626002403/wm4244sup1.cif

Structure factors: contains datablock(s) I. DOI: 10.1107/S2414314626002403/wm4244Isup2.hkl

CCDC reference: 2535729

Additional supporting information:  crystallographic information; 3D view; checkCIF report

## Figures and Tables

**Table 1:** Hydrogen-bond geometry (Å, °)

*D*—H⋯*A*	*D*—H	H⋯*A*	*D*⋯*A*	*D*—H⋯*A*
O2—H2*a*⋯F4^i^	0.852 (18)	1.862 (18)	2.7091 (10)	172 (2)
O2—H2*b*⋯F3	0.91 (2)	1.83 (2)	2.7206 (10)	164 (2)

Symmetry code: (i) 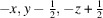.

**Table 2:** Experimental details

Crystal data	
Chemical formula	[Ni(C_10_H_7_NOS)_2_(H_2_O)_2_](BF_4_)_2_
*M*_r_	646.84
Crystal system, space group	Monoclinic, *P*2_1_/*c*
Temperature (K)	100
*a*, *b*, *c* (Å)	7.07483 (19), 11.9935 (3), 15.2159 (4)
β (°)	102.760 (3)
*V* (Å^3^)	1259.22 (6)
*Z*	2
Radiation type	Mo *K*α
μ (mm^−1^)	1.03
Crystal size (mm)	0.26 × 0.17 × 0.13 × 0.10 (radius)

Data collection	
Diffractometer	XtaLAB Synergy, Single source at home/near, HyPix-Arc 100
Absorption correction	Multi-scan (*CrysAlis PRO*; Rigaku OD, 2024 ▸)
*T*_min_, *T*_max_	0.857, 0.858
No. of measured, independent and observed [*I* ≥ 2u(*I*)] reflections	10509, 10509, 9767
*R*_int_	0.048
(sin θ/λ)_max_ (Å^−1^)	0.848

Refinement	
*R*[*F*^2^ > 2σ(*F*^2^)], *wR*(*F*^2^), *S*	0.030, 0.086, 1.04
No. of reflections	10509
No. of parameters	260
No. of restraints	111
H-atom treatment	All H-atom parameters refined
Δρ_max_, Δρ_min_ (e Å^−3^)	1.02, −0.47

Computer programs: *CrysAlis PRO* (Rigaku OD, 2024 ▸), *SHELXS* (Sheldrick, 2008 ▸), *OLEX2.refine* (Bourhis *et al.*, 2015 ▸), *NoSpherA2* (Kleemiss *et al.*, 2021 ▸), *OLEX2* (Dolomanov *et al.*, 2009 ▸), *Mercury* (Macrae *et al.*, 2020 ▸) and *publCIF* (Westrip, 2010 ▸).

## full crystallographic data

### trans-Diaquabis(pyridin-2-yl thiophen-2-yl ketone-κ2N,O)nickel(II) bis(tetrafluoridoborate) Crystal data

**Table:**

[Ni(C_10_H_7_NOS)_2_(H_2_O)_2_](BF_4_)_2_	*F*(000) = 653.806
*M**_r_* = 646.84	*D*_x_ = 1.706 Mg m^−^^3^
Monoclinic, *P*2_1_/*c*	Mo *K*α radiation, λ = 0.71073 Å
*a* = 7.07483 (19) Å	Cell parameters from 13596 reflections
*b* = 11.9935 (3) Å	θ = 2.2–37.0°
*c* = 15.2159 (4) Å	µ = 1.03 mm^−^^1^
β = 102.760 (3)°	*T* = 100 K
*V* = 1259.22 (6) Å^3^	Block, dark yellow
*Z* = 2	0.26 × 0.17 × 0.13 × 0.10 (radius) mm

### trans-Diaquabis(pyridin-2-yl thiophen-2-yl ketone-κ2N,O)nickel(II) bis(tetrafluoridoborate) Data collection

**Table:**

XtaLAB Synergy, Single source at home/near, HyPix-Arc 100 diffractometer	10509 independent reflections
Radiation source: fine-focus sealed X-ray tube, Enhance (Mo) X-ray Source	9767 reflections with *I*≥ 2u(*I*)
Graphite monochromator	*R*_int_ = 0.048
Detector resolution: 10.0000 pixels mm^-1^	θ_max_ = 37.1°, θ_min_ = 2.2°
ω scans	*h* = −11→11
Absorption correction: multi-scan (CrysAlisPro; Rigaku OD, 2024)	*k* = −20→20
*T*_min_ = 0.857, *T*_max_ = 0.858	*l* = −25→25
10509 measured reflections	

### trans-Diaquabis(pyridin-2-yl thiophen-2-yl ketone-κ2N,O)nickel(II) bis(tetrafluoridoborate) Refinement

**Table:**

Refinement on *F*^2^	0 constraints
Least-squares matrix: full	Primary atom site location: structure-invariant direct methods
*R*[*F*^2^ > 2σ(*F*^2^)] = 0.030	All H-atom parameters refined
*wR*(*F*^2^) = 0.086	*w* = 1/[σ^2^(*F*_o_^2^) + (0.0571*P*)^2^ + 0.1359*P*] where *P* = (*F*_o_^2^ + 2*F*_c_^2^)/3
*S* = 1.04	(Δ/σ)_max_ = −0.001
10509 reflections	Δρ_max_ = 1.02 e Å^−^^3^
260 parameters	Δρ_min_ = −0.46 e Å^−^^3^
111 restraints	

### trans-Diaquabis(pyridin-2-yl thiophen-2-yl ketone-κ2N,O)nickel(II) bis(tetrafluoridoborate) Special details

**Table:**

Refinement. NoSpherA2 refinement, some displacement ellipsoid restraints used. Non-merohedral twinning was handled with CrysAlisPro, twin law [-1 0 0 / 0 -1 0 / 0.9467 0 1] and refined twin scale factor 0.4990 (6).

### trans-Diaquabis(pyridin-2-yl thiophen-2-yl ketone-κ2N,O)nickel(II) bis(tetrafluoridoborate) Fractional atomic coordinates and isotropic or equivalent isotropic displacement parameters (Å^2^)

**Table:**

	*x*	*y*	*z*	*U*_iso_*/*U*_eq_	
Ni1	0.0	0.5	0.0	0.01554 (3)	
S1	0.51294 (3)	0.714938 (19)	0.195111 (15)	0.02452 (4)	
O1	0.20854 (7)	0.60036 (5)	0.07412 (4)	0.01866 (9)	
O2	−0.10841 (9)	0.46202 (6)	0.11422 (5)	0.02351 (10)	
H2a	−0.122 (3)	0.3961 (16)	0.1328 (14)	0.051 (4)	
H2b	−0.062 (3)	0.5008 (14)	0.1661 (14)	0.038 (5)	
N2	−0.14605 (7)	0.64682 (5)	−0.00535 (5)	0.01763 (10)	
C1	−0.33236 (9)	0.66168 (7)	−0.04418 (6)	0.02221 (13)	
H1	−0.407 (2)	0.5867 (17)	−0.0779 (14)	0.051 (5)	
C2	−0.42673 (10)	0.76294 (8)	−0.03927 (6)	0.02484 (15)	
H2	−0.5776 (18)	0.7711 (16)	−0.0713 (13)	0.040 (4)	
C3	−0.32397 (11)	0.84908 (7)	0.01029 (7)	0.02557 (15)	
H3	−0.3928 (19)	0.9261 (14)	0.0201 (12)	0.035 (4)	
C4	−0.13057 (10)	0.83255 (6)	0.05346 (6)	0.02240 (13)	
H4	−0.047 (2)	0.8930 (16)	0.0989 (13)	0.043 (4)	
C5	−0.04442 (9)	0.73122 (6)	0.04249 (5)	0.01673 (10)	
C6	0.16137 (8)	0.69937 (6)	0.08269 (5)	0.01650 (10)	
C7	0.30595 (9)	0.77502 (6)	0.13156 (5)	0.01887 (11)	
C8	0.31606 (11)	0.89175 (7)	0.13509 (6)	0.02567 (14)	
H8	0.217 (2)	0.9425 (13)	0.1003 (12)	0.059 (5)	
C9	0.49357 (14)	0.92843 (9)	0.18999 (8)	0.03150 (18)	
H9	0.527 (3)	1.0200 (18)	0.206 (2)	0.080 (9)	
C10	0.61176 (13)	0.84113 (9)	0.22691 (7)	0.03060 (17)	
H10	0.754 (2)	0.8531 (17)	0.2664 (15)	0.059 (6)	
F1	0.16379 (8)	0.59372 (6)	0.40965 (5)	0.03488 (13)	
F2	−0.13872 (8)	0.63709 (9)	0.33296 (5)	0.04540 (19)	
F3	0.08886 (10)	0.58187 (7)	0.25713 (6)	0.04152 (17)	
F4	0.11487 (11)	0.75000 (6)	0.32510 (6)	0.03963 (15)	
B1	0.05459 (10)	0.63984 (7)	0.33141 (6)	0.01851 (12)	

### trans-Diaquabis(pyridin-2-yl thiophen-2-yl ketone-κ2N,O)nickel(II) bis(tetrafluoridoborate) Atomic displacement parameters (Å^2^)

**Table:**

	*U*^11^	*U*^22^	*U*^33^	*U*^12^	*U*^13^	*U*^23^
Ni1	0.01687 (5)	0.01203 (5)	0.01510 (6)	0.00016 (3)	−0.00211 (4)	−0.00188 (4)
S1	0.02372 (7)	0.02472 (9)	0.02122 (9)	−0.00632 (5)	−0.00344 (6)	0.00232 (7)
O1	0.01799 (17)	0.0133 (2)	0.0212 (2)	0.00127 (13)	−0.00320 (15)	−0.00312 (18)
O2	0.0323 (2)	0.0169 (3)	0.0205 (3)	−0.00054 (18)	0.0039 (2)	−0.0011 (2)
H2a	0.081 (11)	0.026 (4)	0.040 (8)	−0.014 (3)	0.002 (6)	0.004 (2)
H2b	0.074 (12)	0.017 (7)	0.018 (5)	−0.007 (5)	−0.001 (4)	0.003 (3)
N2	0.01724 (19)	0.0151 (2)	0.0179 (3)	0.00239 (15)	−0.00166 (16)	0.0000 (2)
C1	0.0179 (2)	0.0233 (3)	0.0222 (3)	0.00361 (19)	−0.0023 (2)	0.0012 (3)
H1	0.036 (7)	0.034 (6)	0.071 (14)	0.002 (3)	−0.013 (6)	−0.014 (4)
C2	0.0200 (2)	0.0272 (4)	0.0264 (4)	0.0085 (2)	0.0031 (2)	0.0062 (3)
H2	0.023 (4)	0.043 (8)	0.050 (10)	0.012 (2)	−0.003 (2)	0.002 (5)
C3	0.0258 (3)	0.0207 (3)	0.0317 (4)	0.0097 (2)	0.0094 (3)	0.0045 (3)
H3	0.031 (5)	0.028 (5)	0.044 (9)	0.014 (2)	0.003 (4)	−0.001 (3)
C4	0.0249 (3)	0.0150 (3)	0.0280 (4)	0.0045 (2)	0.0072 (2)	−0.0007 (3)
H4	0.043 (6)	0.024 (7)	0.056 (10)	0.005 (3)	0.001 (4)	−0.016 (4)
C5	0.0192 (2)	0.0119 (2)	0.0180 (3)	0.00215 (16)	0.00177 (18)	−0.0002 (2)
C6	0.0182 (2)	0.0127 (3)	0.0167 (3)	−0.00050 (16)	−0.00035 (18)	−0.0018 (2)
C7	0.0222 (2)	0.0147 (3)	0.0178 (3)	−0.00397 (17)	0.0005 (2)	−0.0022 (2)
C8	0.0301 (3)	0.0187 (3)	0.0265 (4)	−0.0087 (2)	0.0025 (3)	−0.0015 (3)
H8	0.059 (4)	0.030 (4)	0.074 (10)	−0.0002 (16)	−0.018 (3)	0.003 (2)
C9	0.0372 (4)	0.0239 (4)	0.0328 (5)	−0.0133 (3)	0.0064 (3)	−0.0097 (3)
H9	0.062 (8)	0.027 (2)	0.12 (2)	−0.0111 (14)	−0.039 (6)	−0.0161 (16)
C10	0.0308 (3)	0.0348 (5)	0.0225 (4)	−0.0144 (3)	−0.0020 (3)	−0.0054 (3)
H10	0.042 (3)	0.035 (6)	0.080 (13)	−0.0138 (15)	−0.025 (3)	−0.006 (3)
F1	0.0359 (2)	0.0336 (3)	0.0294 (3)	0.0018 (2)	−0.0050 (2)	0.0100 (3)
F2	0.0210 (2)	0.0763 (6)	0.0379 (4)	−0.0031 (2)	0.0043 (2)	0.0044 (4)
F3	0.0493 (3)	0.0429 (4)	0.0327 (3)	−0.0044 (3)	0.0096 (3)	−0.0207 (3)
F4	0.0557 (3)	0.0152 (3)	0.0485 (5)	−0.0017 (2)	0.0129 (3)	0.0015 (3)
B1	0.0191 (2)	0.0171 (3)	0.0180 (3)	−0.00108 (19)	0.0011 (2)	−0.0020 (3)

### trans-Diaquabis(pyridin-2-yl thiophen-2-yl ketone-κ2N,O)nickel(II) bis(tetrafluoridoborate) Geometric parameters (Å, º)

**Table:**

Ni1—O1^i^	2.0402 (5)	C3—H3	1.070 (15)
Ni1—O1	2.0402 (5)	C3—C4	1.3950 (11)
Ni1—O2^i^	2.0989 (7)	C4—H4	1.082 (17)
Ni1—O2	2.0989 (7)	C4—C5	1.3861 (10)
Ni1—N2^i^	2.0342 (6)	C5—C6	1.4987 (9)
Ni1—N2	2.0342 (6)	C6—C7	1.4445 (9)
S1—C7	1.7243 (7)	C7—C8	1.4022 (11)
S1—C10	1.6924 (9)	C8—H8	0.989 (15)
O1—C6	1.2481 (8)	C8—C9	1.4160 (12)
O2—H2a	0.852 (18)	C9—H9	1.14 (2)
O2—H2b	0.91 (2)	C9—C10	1.3797 (16)
N2—C1	1.3321 (8)	C10—H10	1.060 (15)
N2—C5	1.3565 (9)	F1—B1	1.3833 (11)
C1—H1	1.109 (19)	F2—B1	1.3734 (9)
C1—C2	1.3958 (11)	F3—B1	1.3931 (11)
C2—H2	1.075 (13)	F4—B1	1.3982 (11)
C2—C3	1.3865 (14)		
			
O1^i^—Ni1—O1	180.0	C4—C3—C2	119.48 (7)
O2—Ni1—O1	91.27 (2)	C4—C3—H3	119.1 (9)
O2—Ni1—O1^i^	88.73 (2)	H4—C4—C3	123.0 (9)
O2^i^—Ni1—O1	88.73 (2)	C5—C4—C3	118.66 (8)
O2^i^—Ni1—O1^i^	91.27 (2)	C5—C4—H4	118.2 (9)
O2^i^—Ni1—O2	180.0	C4—C5—N2	121.58 (6)
N2^i^—Ni1—O1^i^	79.07 (2)	C6—C5—N2	112.38 (6)
N2^i^—Ni1—O1	100.93 (2)	C6—C5—C4	125.99 (7)
N2—Ni1—O1	79.07 (2)	C5—C6—O1	117.25 (6)
N2—Ni1—O1^i^	100.93 (2)	C7—C6—O1	118.43 (6)
N2^i^—Ni1—O2^i^	86.89 (3)	C7—C6—C5	124.31 (6)
N2—Ni1—O2	86.89 (3)	C6—C7—S1	116.32 (5)
N2^i^—Ni1—O2	93.11 (3)	C8—C7—S1	111.46 (5)
N2—Ni1—O2^i^	93.11 (3)	C8—C7—C6	132.16 (7)
N2^i^—Ni1—N2	180.0	H8—C8—C7	124.7 (9)
C10—S1—C7	91.87 (4)	C9—C8—C7	111.34 (8)
C6—O1—Ni1	116.21 (4)	C9—C8—H8	123.8 (9)
H2a—O2—Ni1^i^	124.3 (14)	H9—C9—C8	122.7 (11)
H2b—O2—Ni1^i^	118.7 (13)	C10—C9—C8	112.53 (8)
H2b—O2—H2a	103.4 (17)	C10—C9—H9	124.6 (12)
C1—N2—Ni1	125.28 (5)	C9—C10—S1	112.79 (6)
C5—N2—Ni1	114.84 (4)	H10—C10—S1	124.3 (12)
C5—N2—C1	119.58 (6)	H10—C10—C9	122.8 (12)
H1—C1—N2	114.8 (9)	F2—B1—F1	110.16 (8)
C2—C1—N2	122.06 (8)	F3—B1—F1	109.54 (7)
C2—C1—H1	123.1 (9)	F3—B1—F2	110.76 (8)
H2—C2—C1	119.6 (10)	F4—B1—F1	108.43 (7)
C3—C2—C1	118.55 (6)	F4—B1—F2	110.11 (8)
C3—C2—H2	121.8 (10)	F4—B1—F3	107.79 (8)
H3—C3—C2	121.3 (8)		
			
Ni1—O1—C6—C5	3.44 (6)	N2—C5—C6—C7	175.51 (6)
Ni1—O1—C6—C7	−177.61 (5)	C1—N2—C5—C4	1.14 (9)
Ni1—N2—C1—C2	175.01 (7)	C1—N2—C5—C6	179.00 (7)
Ni1—N2—C5—C4	−172.91 (6)	C1—C2—C3—C4	0.11 (10)
Ni1—N2—C5—C6	4.95 (6)	C2—C1—N2—C5	1.63 (10)
S1—C7—C6—O1	−14.34 (7)	C2—C3—C4—C5	2.48 (10)
S1—C7—C6—C5	164.53 (5)	C3—C4—C5—C6	179.26 (7)
S1—C7—C8—C9	−0.05 (7)	C4—C5—C6—C7	−6.74 (10)
S1—C10—C9—C8	0.81 (8)	C5—C6—C7—C8	−18.60 (10)
O1—C6—C5—N2	−5.61 (8)	C6—C7—S1—C10	177.93 (7)
O1—C6—C5—C4	172.14 (7)	C6—C7—C8—C9	−177.03 (10)
O1—C6—C7—C8	162.53 (7)	C7—S1—C10—C9	−0.71 (6)
N2—C1—C2—C3	−2.25 (10)	C7—C8—C9—C10	−0.48 (9)
N2—C5—C4—C3	−3.18 (9)	C8—C7—S1—C10	0.43 (7)

Symmetry code: (i) −*x*, −*y*+1, −*z*.

### trans-Diaquabis(pyridin-2-yl thiophen-2-yl ketone-κ2N,O)nickel(II) bis(tetrafluoridoborate) Hydrogen-bond geometry (Å, º)

**Table:**

*D*—H···*A*	*D*—H	H···*A*	*D*···*A*	*D*—H···*A*
O2—H2*a*···F4^ii^	0.852 (18)	1.862 (18)	2.7091 (10)	172 (2)
O2—H2*b*···F3	0.91 (2)	1.83 (2)	2.7206 (10)	164 (2)

Symmetry code: (ii) −*x*, *y*−1/2, −*z*+1/2.
